# Nanomotion-Based Drug Sensitivity Prediction in Ovarian and Colon Cancer Cell Lines Using Machine Learning

**DOI:** 10.1021/acsptsci.5c00312

**Published:** 2025-08-18

**Authors:** Katja Fromm, Jan Winnicki, Grzegorz Jóźwiak, Gino Cathomen, Christine Wagner, Marta Pla Verge, Eric Delarze, Michał Świątkowski, Grzegorz Wielgoszewski, Maria Ines Villalba, Laura Munch, Sandor Kasas, Danuta Cichocka, Alexander Sturm

**Affiliations:** † 727115 Resistell AG, Hofackerstrasse 40, 4132 Muttenz, Switzerland; ‡ Laboratory of Biological Electron Microscopy (LBEM), 27218 École Polytechnique Fédérale de Lausanne (EPFL), Université de Lausanne, 1015 Lausanne, Switzerland; § Centre Universitaire Romand de Médecine Légale (UFAM), Université de Lausanne, 1000 Lausanne, Switzerland

**Keywords:** nanomotions, cancer drug sensitivity test, colon cancer, doxorubicin, ovarian cancer

## Abstract

Cancer drug resistance remains a critical challenge in oncology, demanding rapid and reliable diagnostic tools to assess tumor cell susceptibility to treatment. This study presents a nanomotion-based drug susceptibility testing (DST) approach, integrating nanoscale movement analysis with supervised machine learning to classify drug-sensitive and drug-resistant cancer cells. Using label-free, real-time nanomotion technology, we measured the dynamic responses of colon cancer (SW480) and ovarian cancer (A2780, A2780ADR) cells to doxorubicin under physiological conditions. Features extracted from nanomotion signals were used to train machine learning models, achieving 90.9% accuracy in distinguishing between doxorubicin-treated and untreated SW480 cells and 84.6% accuracy in classifying doxorubicin-sensitive and -resistant ovarian cancer cells. The model achieved perfect classification of resistant A2780ADR cells in an independent test set after only 4 h and 15 min of exposure to the drug. Unlike genetic tests that infer drug resistance from molecular markers or metabolic assays requiring extended incubation times, nanomotion-based DST provides a direct phenotypic readout, offering a faster, label-free alternative for assessing tumor cell responses. While further dataset expansion and model refinement are necessary to enhance generalizability, these results underscore the potential of nanomotion technology as a rapid, phenotypic DST for personalized oncology. By directly measuring the mechanical behavior of cancer cells in response to chemotherapy, this method could transform clinical decision-making, enabling faster, more precise treatment strategies to combat drug resistance in cancer.

## Graphical abstract

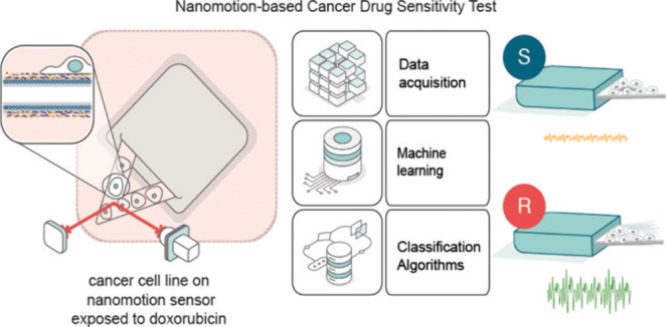

Cancer remains a major global health challenge and one of the leading causes of mortality worldwide.^1,2^ Despite significant advances in precision medicine, standard treatments in daily clinical practice still rely heavily on cytotoxic agents, which often lead to drug resistance and suboptimal therapeutic outcomes.^3,4^ Approximately 90% of cancer-related deaths are associated with drug resistance.^5^ The ability of cancer cells to develop resistance over time presents a critical obstacle in effective cancer treatment.^6^ To improve therapeutic efficacy and ensure optimal patient-specific treatment, there is a growing need for personalized drug sensitivity testing (DST) that can predict a patient’s response to chemotherapy.^7–9^

Nanomotion technology has emerged as a promising approach to assess cellular viability and drug response in real time.^10–20^ Previous studies have demonstrated the applicability of nanomotion-based DST for cancer cells, providing a rapid and label-free method to evaluate treatment efficacy.^12,13^ This technology detects nanoscale movements generated by living cells, which can be modulated by drug exposure. For that purpose, cells are attached to a cantilever, a miniature springboard of approximately 100 μm in length, which captures nanoscale movements of cells that are exposed to chemotherapeutics.^13^ This technology originates from atomic force microscopy and has been intensively investigated for antibiotic susceptibility testing for bacteria.^10,11^

One chemotherapeutic agent widely used in clinical oncology is doxorubicin (doxorubicin, PubChem CID 31703, Suppl. Figure S1), an FDA-approved anthracycline for the treatment of cancer. Doxorubicin intercalates into DNA, inhibiting cell proliferation by blocking the activity of topoisomerase II and thereby also generating free radicals that cause oxidative damage to cellular structures.^21–23^ Despite its efficacy in treating various malignancies, doxorubicin is associated with significant side effects and the emergence of resistance, necessitating patient-specific sensitivity assessments before administration.^24,25^ By integration of nanomotion-based DST with machine learning (ML) models, it is possible to develop a robust classification system capable of distinguishing between drug-sensitive and drug-resistant cancer cells, enabling a more personalized treatment approach based on diagnostic results. Such an approach could enable more precise treatment decisions by comparing the response of cancerous and healthy cells. A similar approach based on nanomotion recordings and ML algorithms has been described for antibiotic susceptibility testing of common bacterial pathogens in sepsis.^10^

This study aimed to advance nanomotion-based DST by incorporating ML techniques to refine classification models for drug responsiveness. We aimed to develop an optimized, personalized DST framework that can guide clinicians in selecting the most effective treatment strategies while minimizing adverse effects. By leveraging the unique biophysical properties of cancer cells and their differential response to chemotherapy, this approach holds the potential to significantly improve patient outcomes and contribute to the evolution of precision oncology.

## Results

### Nanomotion Recording in CO_2_-Supplemented Incubator

A novel experimental setup was developed to maintain optimal culture conditions for cancer cells during nanomotion recording. This nanomotion measurement system was designed to ensure that cells were cultured at 37 °C in a 5% CO_2_ environment, thereby preserving physiological conditions throughout the measurement process. The measurement head, a vibration isolation system, and a specially designed holder plate were placed inside a CO_2_-supplied incubator, while the supporting electronics, light source, and photodetector remained outside (Suppl. Figure S2). This setup enables nanomotion measurements of mammalian cells under physiological conditions while still providing readouts comparable to those previously obtained with bacteria.^10^

Prior to the experiment, cancer cells were cultured in a medium optimized for the specific cell line, and the same medium was maintained during the nanomotion measurements. For the nanomotion recording, triangular cantilevers with a surface area of 18,700 ± 500 μm^2^ were employed, providing a sufficiently large surface area for the attachment of human cancer cells. After baseline measurements of the bare cantilever, cells were allowed to attach, and their nanomotions were subsequently measured in culture medium for 120 min. Doxorubicin was then added to the medium inside the measurement chamber, and the recording continued for 2–6 h, depending on the cell line.

In contrast to previous studies, where cells were exposed to nonphysiological conditions, such as room temperature,^13^ our approach maintained cells under physiological conditions throughout the entire experiment. The process of cell attachment was simplified in this setup, as the cantilever was no longer used to “fish” the cells for attachment, which required finicky operations under the microscope and thus elapsed hands-on time.^13^ In this study, cells were allowed to settle directly onto a functionalized cantilever for 90 min, significantly simplifying the protocol and increasing the number of cells attached to the cantilever as a preparation for the nanomotion recording in the measurement chamber (Figure 1a–c). Although the duration of the nanomotion experiments varied depending on the specific cell line investigated, the total recording of attached cells generally did not exceed 8 h. The data obtained from these nanomotion measurements were then used to generate the first classification models for a nanomotion-based DST in cancer cell lines.

**Figure 1:**
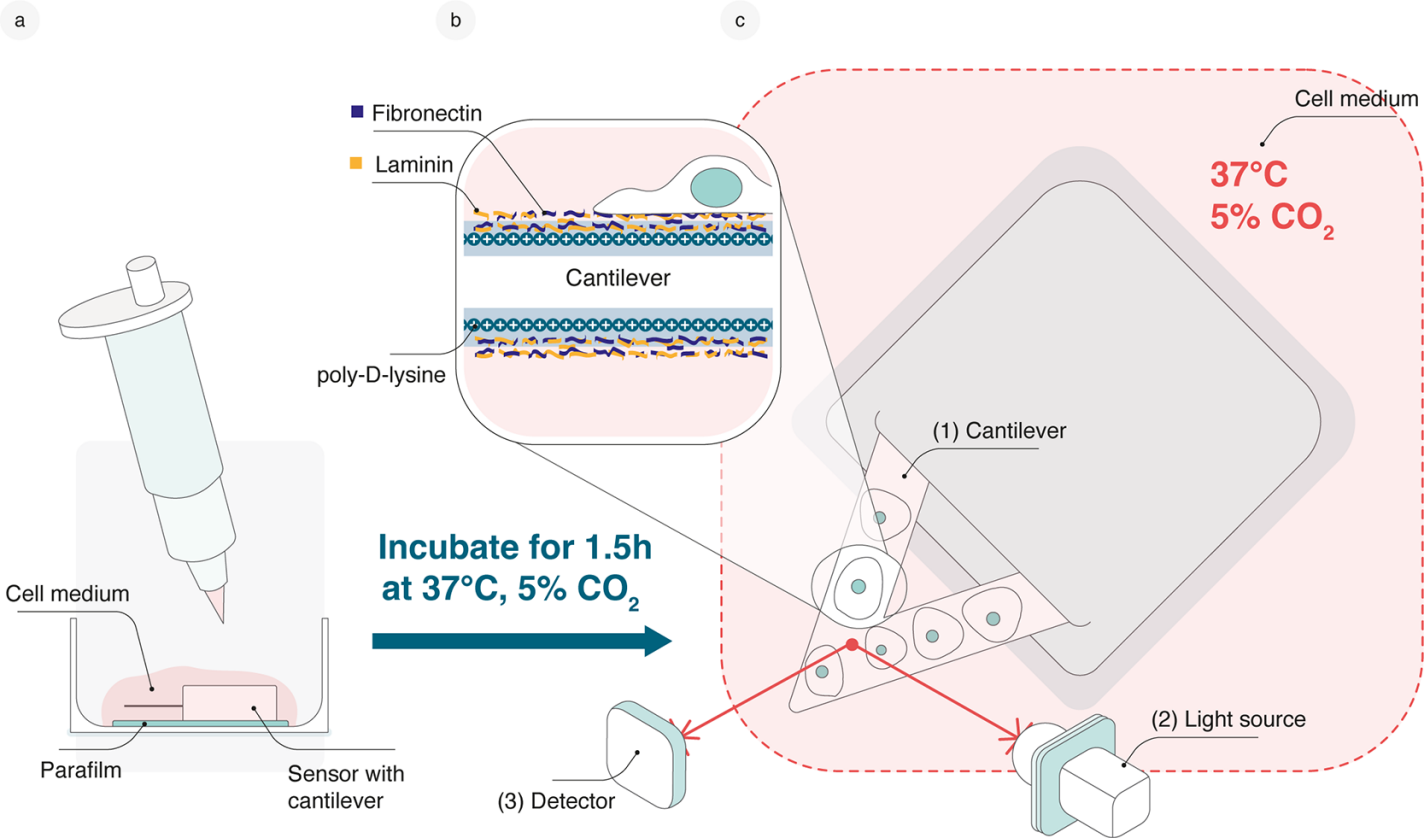
Experimental setup of nanomotion experiments. (a) Functionalized cantilever sensor is placed on parafilm inside a Petri dish. The cell suspension is spread over the sensor, and cells are incubated for 90 min at 37 °C and 5% CO_2_ to ensure stable attachment before transferring the sensor to the measurement chamber of the nanomotion device. (b) Schematic illustration of cancer cells attached to a cantilever that has been sequentially functionalized with positively charged poly-d-lysine, followed by the extracellular matrix components laminin and fibronectin. (c) Schematic representation of the nanomotion measurement setup: (1) Cancer cells are attached to the cantilever. (2) A superluminescent light-emitting diode (SLED) is directed at the tip of the triangular cantilever. (3) A photodetector captures the deflection signals. The measurement head, containing the measurement chamber, is placed inside an incubator to maintain optimal experimental conditions (37 °C, 5% CO_2_). The light source, photodetector, and supporting electronics remain outside the incubator (see also Suppl. Figure S2).

### Optimization of Attachment on Functionalized Cantilevers for Several Cancer Cell Lines

To optimize stable cell attachment to the cantilever during nanomotion experiments, various linking agents were investigated for their ability to facilitate cell adhesion. Poly-d-lysine (PDL) has been widely used in bacterial studies to establish stable interactions between the negatively charged bacterial surface and the positively charged PDL-coated cantilever.^10^ While this method was effective in bacterial systems, we observed that PDL did not facilitate stable attachment of human cancer cells within 90 min, as indicated by their round, nonphysiological shape. Specifically, cells from the colon cancer cell line SW480 and the ovarian cancer cells A2780 maintained a rounded shape that was uncharacteristic of their typical, attached shape (Figure 2a–c). Therefore, alternative attachment facilitators were researched to provide a more stable cell attachment.

**Figure 2:**
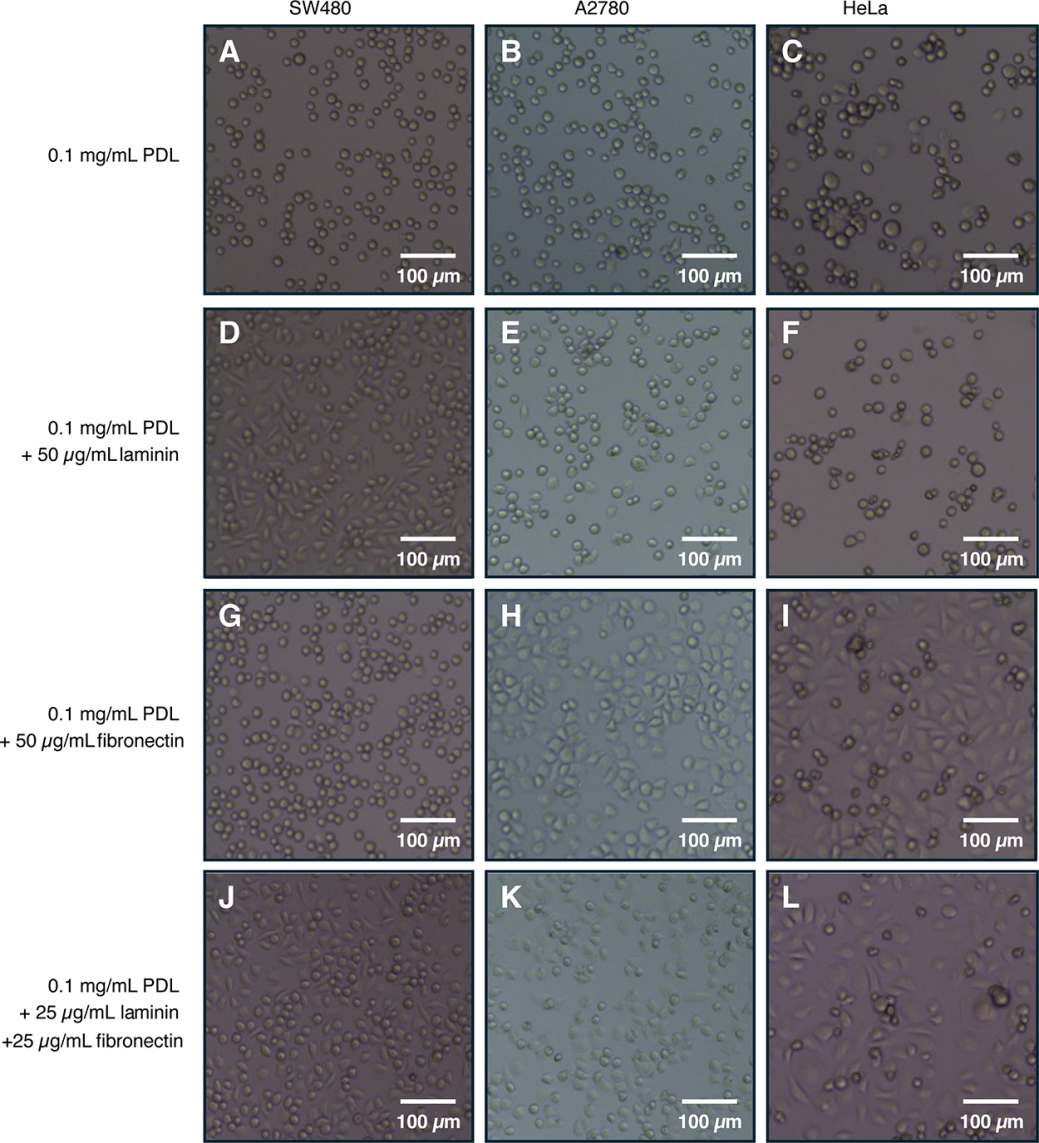
Functionalization of glass surfaces with PDL + laminin + fibronectin enables stable cell attachment. The ovarian cancer cell line A2780 was cultured in RPMI + 10% FCS, while the colon cancer cell line SW480 and the cervical cancer cell line HeLa were cultured in DMEM + 10% FCS. All cells were maintained at 37 °C and 5% CO_2_. A total of 5 × 10^4^ cells were seeded per well in a 96-well plate, incubated for 1 h, and subsequently washed with PBS to remove nonattached cells. After washing, 100 μL of fresh cell culture medium was added to each well, and cell attachment was assessed using microscopy. (A–L) Representative microscopy images of cell attachment on different surface coatings: (A–C) Poly-d-lysine (PDL) (0.1 mg/mL) functionalization: (A) SW480, (B) A2780, and (C) HeLa cells. (D–F) PDL (0.1 mg/mL) + laminin (50 μg/mL) functionalization: (D) SW480, (E) A2780, and (F) HeLa cells. (G–I) PDL (0.1 mg/mL) + fibronectin (50 μg/mL) functionalization: (G) SW480, (H) A2780, and (I) HeLa cells. (J–L) PDL (0.1 mg/mL) + laminin (25 μg/mL) + fibronectin (25 μg/mL) functionalization: (J) SW480, (K) A2780, and (L) HeLa cells. All images are representative of three independent experiments.

To assess the effectiveness of different linking agents, untreated 96-well plates with a glass surface were coated with various functionalization agents. The glass-bottom plates were chosen to mimic the conditions on the silicon cantilevers used during nanomotion measurements. We utilized the same attachment procedures as for cell attachments on cantilevers, followed by nanomotion measurements. In brief, after functionalization of the glass surface, the cell suspension was layered on top and incubated for 90 min at 37 °C and 5% CO_2_. To remove dead or nonattached cells, the suspension was discarded, and the surface was gently washed with PBS. Cell culture medium was added, and the cell attachment was assessed.

The ability of the cells to adhere to the glass surface was tested using two different approaches: a quantitative approach by performing a resazurin assay and a qualitative microscopic approach. To identify linking agents suitable for cancer cells of various tissues, three different cancer cell lines—SW480 (colon cancer), HeLa (cervical cancer), and A2780 (ovarian cancer)—were investigated for cell attachment on the functionalized surfaces.

A resazurin assay was used for quantitative analysis. Resazurin is reduced to resorufin by living cells, indicating cell viability. In this setup, the ratio between resazurin and resorufin also indicates increased cell attachment depending on the used linking agent, as higher numbers of attached cells lead to greater substrate conversion. The mean fluorescence intensity (MFI) of resorufin was measured, with increased MFI indicating a higher number of attached cells. In general, the results demonstrated that cell attachment was significantly enhanced on functionalized surfaces compared to untreated ones (Suppl. Figure S3). However, the tested cell lines appeared to attach with varying affinities, depending on the linking agents used. For instance, treatment with PDL alone led to increased MFIs across all cell lines. In contrast, coating with MAPTrix significantly elevated MFI values compared to the untreated surface only in HeLa and SW480 cells but not in A2780 cells.

Similarly, higher MFI values were observed on fibronectin-coated surfaces for A2780 and HeLa cells, while laminin-coated surfaces resulted in increased MFI values for SW480 cells. Overall, glass surfaces treated with the extracellular matrix (ECM) component preferred by each cell line—either laminin or fibronectin—along with PDL, showed the highest MFI values.

These findings suggest that a combination of nonspecific charge-dependent interactions and specific binding to ECM components promotes the most stable cell attachment. The use of PDL combined with laminin and fibronectin led to elevated MFI values across all three cell lines compared with the uncoated surface, potentially indicating a broadly applicable strategy for stable cell attachment, regardless of tissue origin.

Further investigation of cell attachment was conducted using microscopy to evaluate the attachment quality, focusing on the cell shape. The same functionalization conditions were employed as those in the quantitative resazurin approach. We observed lower cell numbers and a nonphysiological, rounded cell shape on surfaces treated only with PDL, MAPTrix, or left untreated (Figure 2 and Suppl. Figure S4). Higher cell numbers and a physiological, spreading cell shape were observed when the glass surface was treated with linking agents, representing ECM components, though with cell line-specific variations (Figure 2 and Suppl. Figure S4). A2780 and HeLa cells showed increased cell numbers and a spreading morphology on surfaces coated with laminin (or PDL + laminin). In contrast, a rounded cell shape was more prevalent on surfaces coated with fibronectin (or PDL + fibronectin). These phenotypes were reversed in SW480 cells, which exhibited a spreading morphology on fibronectin-coated surfaces (or PDL + fibronectin). Notably, surfaces treated with a combination of PDL, laminin, and fibronectin supported high cell numbers and a spreading phenotype across all three cell lines (Figure 2). Together, the results from the quantitative resazurin-based assay and microscopic analysis indicate that stable cell attachment is promoted by the interaction between the positively charged PDL and the negatively charged cell surface, along with specific binding to the ECM components laminin and fibronectin.

### Classification Model for Drug Sensitivity in Colon Cancer Cells

Before developing a classification model capable of differentiating between drug-sensitive and drug-resistant cells, we initially implemented a model to successfully discriminate between untreated and doxorubicin-treated SW480 colon cancer cells.

SW480 cells were attached to fibronectin-coated cantilevers and placed in the measurement chamber for nanomotion recording. Nanomotion measurements were performed during a 2 h incubation period in DMEM medium, followed by an additional 2 h in either medium alone or medium supplemented with 32 μM doxorubicin (Figure 3a, b). This relatively high concentration of doxorubicin was selected based on previously reported IC_5__0_ values for doxorubicin-resistant SW480 cells, which range around 32 μM after 24–48 h of incubation.^26,27^ Accordingly, we used 32 μM doxorubicin for the two h drug exposure phase to enable clear differentiation between doxorubicin-treated and untreated SW480 cells. For each experiment the attachment was monitored before the measurement and at the end of drug exposure by taking a microscopy picture of the cantilever (Figure 3c).

**Figure 3:**
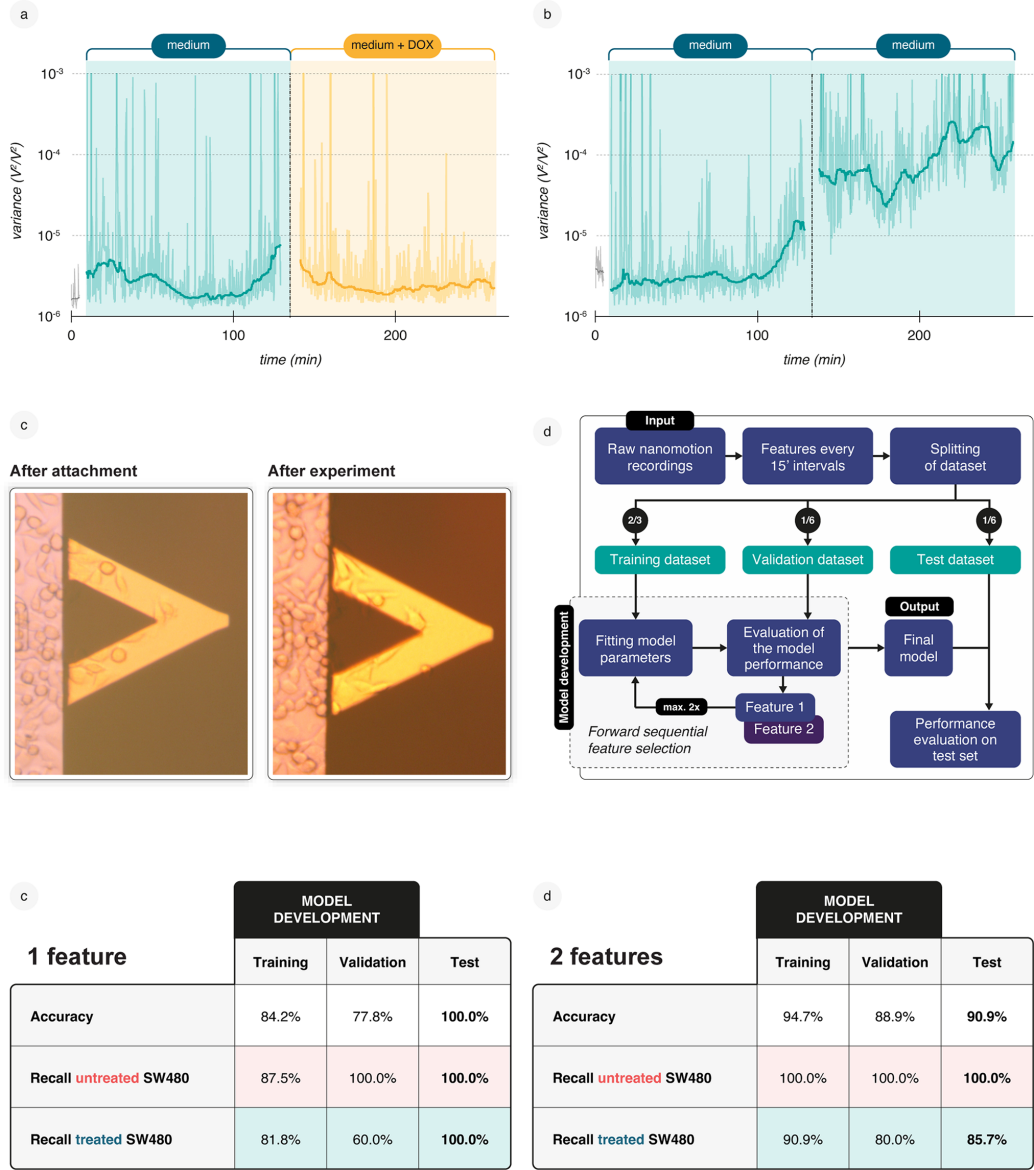
Machine learning (ML) assisted model discriminates between doxorubicin-treated and nontreated colon cancer cells SW480 with high accuracy. The doxorubicin-susceptible colon cancer cell line SW480 was cultured in DMEM supplemented with 10% heat-inactivated FCS at 37 °C and 5% CO_2_. Cells were detached using trypsin-EDTA, resuspended in DMEM + 10% FCS, and attached to the functionalized microcantilever. All nanomotion experiments were conducted in the same medium. (a, b) Three distinct signal recording phases were performed: (i) *Blank* phase—Deflection of bare cantilevers was measured (gray). (ii) *Medium* phase—Deflections of a cantilever with attached SW480 cells were recorded (green). (iii) *Drug* phase–The cantilever deflection was measured after (a) exposure to 32 μM doxorubicin or (b) a second *Medium* phase without drug addition. The variance of the cantilever deflections was calculated every 8 s. (c) Microscopic images of SW480 cells attached to a cantilever functionalized with 50 μg/mL fibronectin were taken immediately after attachment and after nanomotion recording. (d) An ML classification model was developed using 58 nanomotion experiments (34 with doxorubicin exposure, 24 without). The data set comprised 34 treated (positive) and 24 untreated (negative) cells, split into training (22 treated and 16 untreated), validation (5 treated and 4 untreated), and test sets (7 treated and 4 untreated), ensuring a balanced representation of the two conditions in each subset. Feature selection and weighting were applied for logistic regression, limiting the model to two features to prevent overfitting. (e) Performance of the best two-feature-based ML model on training, validation, and test data sets using one feature and (f) based on two features.

In order to train the algorithm, a total of 58 experiments were conducted, comprising 24 experiments with untreated cells and 34 experiments with cells treated with 32 μM doxorubicin. The resulting data were included in the analysis. Each recording was acquired at a sampling frequency of 60 kHz, thereby capturing both low- and high-frequency vibrations. The acquired signal was transformed into a set of meaningful features that encapsulated information regarding both the cells and the measurement environment. To achieve this, a modified version of the Welch algorithm^28^ for obtaining the power spectral density was applied to 5 min time intervals.

Rather than computing the mean of periodograms, quantile spectra^29^ were employed to represent the 10th to 90th percentiles of the spectrum for each 5 min recording period. From these quantile spectra, features were derived that described the relationships between quantiles across specific frequency ranges and 10 min intervals. This approach yielded several hundred thousand quantile features from each recording. Subsequently, a feature selection algorithm was applied to identify the most dominant features for discriminating between treated and untreated cells, but considering the limited data set size, the model contained not more than two features.

In addition, for an unbiased performance evaluation, the dataset was partitioned into training, validation, and test sets in a 4:1:1 ratio. The model was fitted by using the training set, and the combined training and validation sets were utilized during the feature selection process. Notably, the nanomotion recordings from the test set were excluded from model development, ensuring an independent assessment of performance (Figure 3d).

Logistic regression was then utilized for classification. Notably, a feature extracted from the low-frequency range (0–10 Hz) in the 10th percentile of the spikiness of the quantile spectrum, observed during the 120–130 and 210–220 min intervals, provided significant discriminatory power. These intervals corresponded to the onset of doxorubicin exposure and a late phase (20–30 min before the recording ended) for the untreated and drug-exposed conditions, respectively.

Using this feature, the model achieved a training accuracy of 84.2% and a testing accuracy of 100%. In this context, accuracy is defined as the proportion of correctly classified samples (both treated and untreated) relative to the total number of samples (Figure 3e). By increasing the information density and incorporating an additional feature into the logistic regression model, accuracy improved to 94.7%, with perfect recall, i.e., concordance, for untreated samples and a recall of 90.9% for doxorubicin-treated samples in the training dataset. This performance was mirrored in the test dataset (90.9% accuracy). The second feature also leveraged low-frequency information (10–100 Hz), specifically the 95th percentile of the Jensen–Shannon distance^30^ between the quantile spectrum and the theoretical chi-squared distribution, computed over similar time intervals (130–140 and 190–200 min; see Figure 3f). Although the testing performance with two features was lower than that with a single feature, it exhibited greater concordance with the training performance, suggesting improved model generalizability and reliability. Average precision (AP) scores were well above the random classifier baseline of 0.64 for the test set, with values approaching 1.0 (Suppl. Figure S5). In conclusion, this demonstrates that minimal feature sets can reliably distinguish between treated and untreated cells.

### DST for Ovarian Cancer Cells

We continued our ML-assisted DST development and tested whether a model could not only discriminate between treated and nontreated cells but also distinguish between susceptible and resistant cells in response to doxorubicin exposure. For this matter, we used the parental ovarian cancer cell line A2780 and its doxorubicin-resistant offspring cell line A2780ADR whose doxorubicin resistance is based on the overexpression of the P-glycoprotein transporter.^31^ To identify a suitable doxorubicin concentration to train the algorithm, we first utilized a resazurin-based cell viability assay to calculate the IC_50_ values for both cell lines. A2780 and A2780ADR cells were seeded in 96-well plates and treated for 24 h with increasing doxorubicin concentrations. Following incubation, the medium was supplemented with 0.005% resazurin, and the relative cell viability was quantified by measuring the absorption of the substrate resazurin (600 nm) and the product resorufin (570 nm) after 4 h using the FLUOstar Omega plate reader. Cytotoxicity was expressed as the relative viability (percentage of control). This method calculated the following IC_50_ values: 0.602 μM for doxorubicin in A2780 and 6.189 μM for doxorubicin in A2780ADR (Suppl. Figure S6). Both ovarian cancer cell lines were attached to cantilevers functionalized with PDL, laminin, and fibronectin. During nanomotion recordings, we observed a lower variance in the ovarian cancer cells compared to the SW480 cells (Figures 3a, b and[Fig fig4]a, b), which could be attributed to differences in their cell properties. As we were initially uncertain about the optimal response time to explore the impact of doxorubicin exposure, we extended the drug exposure from 2 to 6 h, increasing the total recording time to 8 h (Figure 4a, b).

**Figure 4:**
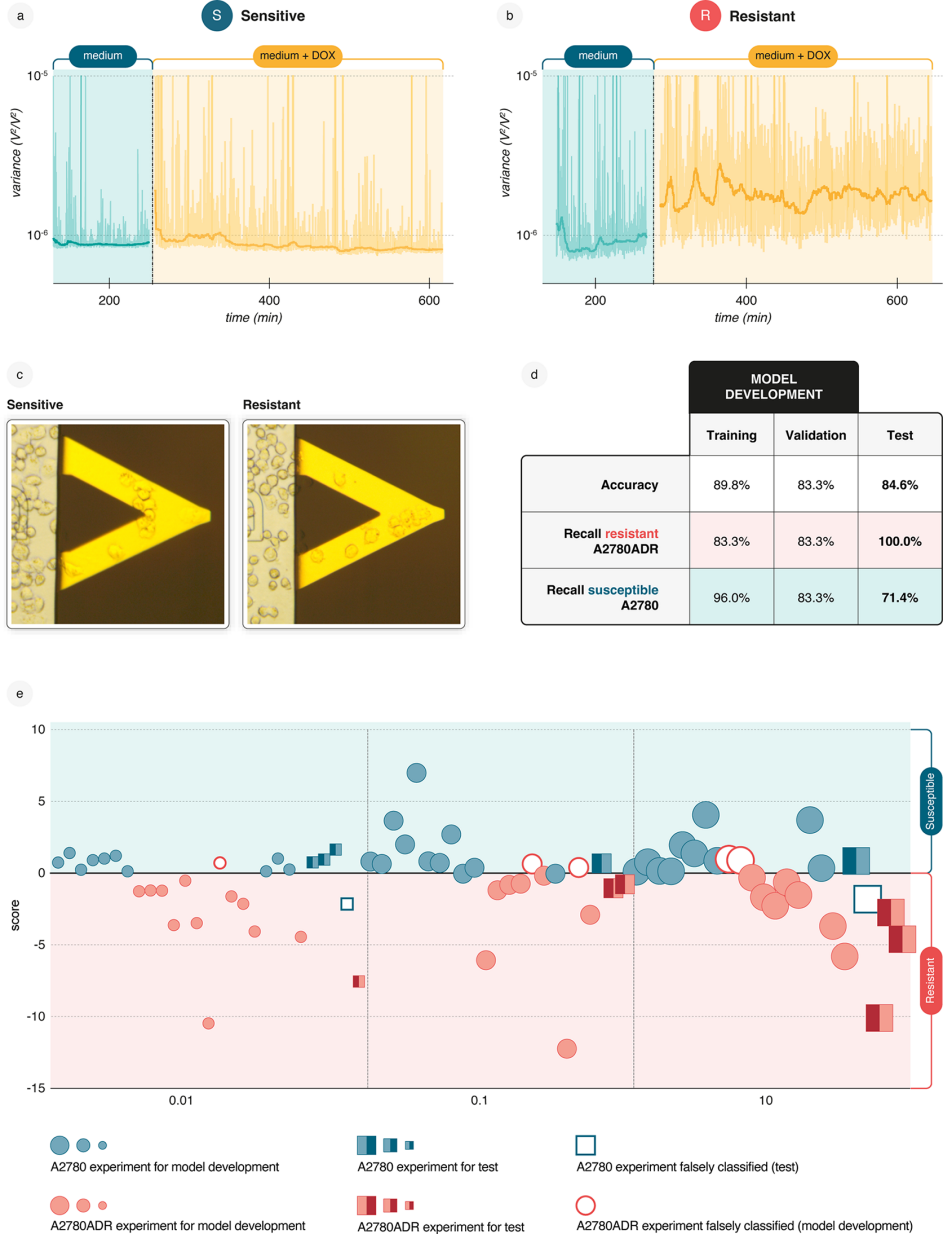
Two-feature-based classification model differentiates between doxorubicin-sensitive and -resistant cells. The ovarian cancer cell lines A2780 (doxorubicin-susceptible) and A2780ADR (doxorubicin-resistant) were cultured in RPMI medium supplemented with 10% heat-inactivated FCS at 37 °C and 5% CO_2_. Cells were detached using trypsin-EDTA and resuspended in RPMI + 10% FCS before being attached to the functionalized cantilever for nanomotion experiments, which were conducted in the same medium. (a, b) Representative 8 h nanomotion recordings showing a 2 h *Medium* phase, followed by a 6 h *Drug* phase with 10 μM doxorubicin. (a) Response of the susceptible A2780 cells, and (b) response of the resistant A2780ADR cells. (c) Microscopy images of attached cells before nanomotion recordings. (d) Machine learning (ML) classification model was developed using 38 susceptible (positive, A2780) and 36 resistant (negative, A2780ADR) cells, divided into training (25 susceptible and 24 resistant), validation (6 susceptible and 6 resistant), and test sets (7 susceptible and 6 resistant). The dataset was split into subsets according to Figure 3d, ensuring a balanced representation of both cell lines and the tested doxorubicin concentrations (0.01 μM, 0.1 μM, and 10 μM). The performance of the best two-feature-based ML model on training, validation, and test data sets is indicated. (e) Classification scores for the 74 nanomotion experiments used in model development (circles) and testing (squares). A classification score ≥ 0 indicates susceptibility, while a score <0 indicates resistance. Susceptible A2780 cells are shown in blue, and resistant A2780ADR cells are shown in red. Misclassified samples are represented by white circles (training) or white squares (test set). The increasing size of the symbols correlates with the higher concentration of doxorubicin.

In the ovarian cancer dataset, 36 resistant (A2780ADR) and 38 susceptible (A2780) samples were analyzed. Our methodology was modified to employ 15 min intervals throughout the drug exposure period for the computation of quantile features for each interval. The medium phase was excluded from feature definition and retained solely for biological considerations (i.e., permitting cellular recovery following attachment stress).

To determine the optimal duration of drug exposure for classification, we evaluated logistic regression models with one or two features within a cross-validation framework, aiming to identify the point at which the performance plateaued. The results demonstrated that accuracy plateaued at 84% after 4 h and 15 min of drug exposure, indicating that the final 1 h and 45 min of nanomotion recording did not enhance model performance (Suppl. Figure S7).

For an unbiased performance evaluation, the dataset was again partitioned into training, validation, and test sets in a 4:1:1 ratio. Again, the nanomotion recordings from the test set were excluded from model development for an unbiased performance assessment. Under this approach, the training accuracy reached 89.8% with a recall of 96.0% for the sensitive A2780 cells, whereas the test accuracy was 84.6% with a recall of 100% for the resistant cells. Overall, the performance observed during cross-validation closely paralleled that of the independent test set (Figure 4d).

The two features employed in this analysis differed from those used for the SW480 cells and were derived from higher frequency ranges (400–1000 Hz and 7000–8000 Hz, just below the cantilever’s resonant frequency). Both features were extracted from the time window spanning 165 to 240 min after the initiation of cell recording. The logistic regression model assigned scores to each nanomotion recording, with scores ≥ 0 corresponding to susceptible classifications and scores <0 corresponding to resistant classifications. Classification scores are presented for each experiment across the different doxorubicin concentrations used (Figure 4e). The precision–recall curves show that the AP consistently exceeds the random baseline of 0.54 for the test set and approaches 0.9, indicating strong predictive accuracy. These results demonstrate that the models are capable of effectively distinguishing between drug-susceptible and resistant cells, achieving a favorable balance between sensitivity and specificity even with minimal feature input (Suppl. Figure S8).

## Discussion

This study demonstrates the potential of nanomotion analysis combined with supervised ML to assess cancer cell susceptibility to doxorubicin. By leveraging nanomotion measurements, we achieved promising results in predicting the cell response to the drug. This approach, building on the success of nanomotion-based techniques in antimicrobial susceptibility testing (AST), suggests a viable path for applying this method to cancer therapeutics.

A key advantage is the use of supervised ML models that focus on features derived from the nanomotion signal. While the biological basis of these features remains unclear, they are fixed in the classification model once developed, relying on consistent signal components rather than black-box models that can complicate regulatory approval. This ensures the development of accurate, interpretable models that are clinically applicable.

Although the dataset was small, this study represents an important step toward developing a reliable method for drug susceptibility testing. Expanding the dataset with more diverse clinical cell samples will improve model performance and generalizability.

While developing a clinically applicable DST is resource-intensive, it offers significant benefits. With over 50% of cancer treatments failing, personalized susceptibility tests could reduce treatment failures, guide informed decisions, and ultimately improve patient outcomes while cutting unnecessary costs.^32^

Nanomotion analysis also provides a direct phenotypic readout, unlike genetic tests or sequencing, which rely on indirect inference of genetic markers. It offers real-time, label-free observations of cellular behavior, making it a faster and more immediate method compared to metabolic markers, such as the resazurin assays presented here or other methodologies.^33–35^ Genetic tests, though valuable, do not assess the immediate effects of the drug on cell behavior, which is complicated by the numerous and diverse multidrug resistance (MDR) mechanisms.^36^ Also, in the case of A2780ADR, adaptive phenotypic responses are involved in its decreased sensitivity toward doxorubicin based on overexpression of the P-glycoprotein transporter.^31^

## Conclusions

While further development is needed, nanomotion analysis combined with supervised ML holds promise for advancing personalized cancer treatment strategies. It offers a faster, more direct alternative to traditional methods in returning phenotypic DST results in only 4 h 15 min of doxorubicin exposure. In the future, it could provide a clinically actionable tool for accelerated decision-making in cancer therapy.

## Materials and Methods

### Cell Lines and Culture Conditions

The colon cancer cell line SW480 (obtained from Sandor Kasas, EPFL) and HeLa cells (ATCC CCL-2, 70037076) were cultured at 37 °C and 5% CO_2_ in DMEM Glutamax (Bioconcept, 1-26F50-I) supplemented with heat-inactivated Fetal Calf Serum (FCS, Bioconcept, 2-01F10-I). The ovarian cancer cell lines A2780 (Sigma/Merck, 21C003) and A2780ADR (Sigma/Merck, 14G008) were cultured in RPMI 1640 supplemented with l-glutamine (Bioconcept, 1-41F03-I) and 10% heat-inactivated FCS.

### Surface Functionalization for Attachment Tests in 96-Well Plates

To facilitate a strong attachment to the cantilever during the experiments, the custom-made sensors, which contain quartz-like tipless cantilevers with a gold coating (Bruker, NPO-10.B), need to be functionalized. To identify the functionalization agent or a mix of different components, which enables the most stable attachment, we functionalized the surface of untreated 96-well plates with a glass bottom, which supposedly mimics the quartz-like cantilever. For surface functionalization, the following agents were tested alone or in a mixture: Poly-d-Lysine hydrobromide (PDL, VWR, ICNA0215017580), MAPTrix Reagent (Sigma/Merck, 160022K), fibronectin from human plasma (Sigma/Merck, FIBRP-RO), and murine laminin (Sigma/Merck, L2020-1MG). Wells were functionalized by incubation with 0.1 mg/mL PDL (w/v in molecular biology-grade water) or 50 μg/mL fibronectin (w/v in molecular biology-grade water) for 20 min at room temperature (RT), 0.1 mg/mL MAPTrix (v/v in sodium bicarbonate pH 8.4–8.6) for 1 h at RT, or 50 μg/mL laminin (w/v in PBS) for 30 min at 37 °C. Following incubation, the solutions were removed, and the wells were gently washed with molecular biology-grade water. The wells were allowed to dry for 15 min. For functionalization with PDL, a sequential approach of fibronectin and laminin was applied. After incubation with 0.1 mg/mL PDL as described, 50 μg/mL fibronectin or 50 μg/mL laminin or a mixture of 25 μg/mL fibronectin and 25 μg/mL laminin were layered on top and incubated for 30 min at 37 °C. Following incubation, the wells were washed with molecular biology-grade water and allowed to dry for 15 min at RT.

### Resazurin-Based Quantification of Cell Attachment

To assess the cell attachment to glass surfaces functionalized with different linking agents, we utilized a resazurin-based quantification assay. Resazurin is reduced to resorufin by the aerobic respiration of metabolically active cells and can be used as an indicator of cell activity. Resazurin is weakly fluorescent and has a blue color (absorbance 600 nm), while resorufin is highly fluorescent and has a pink color (absorbance 570 nm). To obtain a cell suspension for attachment tests, the culture medium was removed, and the cells were washed with PBS (VWR, 786-027). 1x Trypsin-EDTA (1x, Sigma/Merck, T4174-100 ML, v/v in PBS) was added and incubated for 30–90 s to detach the cells. Cells were collected in their corresponding culture medium. 5 × 10^4^ cells per well were seeded into the functionalized 96-well plate and incubated for 120 min at 37 °C and 5% CO_2_. The supernatant was removed, and the cells were gently washed with PBS to remove nonattached cells. After the washing step, a fresh cell culture medium was added. Subsequently, the medium was supplemented with 0.005% resazurin (w/v in ddH_2_O, VWR, 199303–1G), and the fluorescence was measured with an excitation maximum at 544 nm and an emission maximum at 590–610 nm every 20 min for 4 h using the FLUOstar Omega plate reader (BMG Labtech).

### Resazurin-Based Determination of Reference Half Maximal Inhibitory Concentration (IC_50_)

To assess the sensitivity of A2780 and A2780ADR cells to doxorubicin, the IC_50_ was determined using a resazurin-based cell viability test. 2 × 10^4^ cells per well were seeded in a black 96-well plate (Huberlab, 7.655 906) and incubated for 24 h. Subsequently, doxorubicin was added at various concentrations. At 24 h after treatment, the medium was supplemented with 0.005% resazurin (w/v in ddH_2_O, VWR, 199303–1G). To quantify the relative cell viability, the absorption of the substrate resazurin (600 nm) and the product resorufin (570 nm) were measured every 20 min for 4 h using the FLUOstar Omega plate reader (BMG Labtech). Cytotoxicity was expressed as relative viability (percentage of control). The percentage of cell survival in the negative control (without doxorubicin treatment) was considered 100. The relative viability was calculated using the following formula:

$$\frac{{\mathrm{Abs}}_{\exp\mathrm{eriment}}-{\mathrm{Abs}}_{\mathrm{background}}}{{\mathrm{Abs}}_{\mathrm{control}}-{\mathrm{Abs}}_{\mathrm{background}}}\times 100$$

The IC_50_ values of doxorubicin for both cell lines were calculated by using GraphPad Prism by applying a variable slope model.

### Cantilever Functionalization

To facilitate cell attachment and prevent detachment during DST recordings, cantilevers (Bruker, NPO-10.B) were sequentially functionalized with Poly-d-Lysine hydrobromide (PDL, VWR, ICNA0215017580), fibronectin (Sigma/Merck, FIBRP-RO), and laminin (Sigma/Merck, L2020-1MG) (Figure 1b). The cantilevers were incubated with 0.1 mg/mL PDL (w/v in molecular biology-grade water) for 20 min at RT. Following incubation, the PDL solution was removed, and the cantilever tips were gently washed with molecular biology-grade water. The cantilevers were allowed to dry for 15 min before applying a mixture of 25 μg/mL fibronectin (w/v in molecular biology-grade water) and 25 μg/mL laminin (w/v in PBS). After 30 min of incubation at 37 °C, the mixture was removed and the cantilevers washed with PBS before being allowed to dry for another 15 min before use.

### Attaching Cells to the Cantilever

SW480, A2780, and A2780ADR cells were cultured as described previously. To obtain a cell suspension for nanomotion recordings, we removed the culture medium and washed the cells with PBS (VWR, no. 786-027). 1x Trypsin-EDTA (Sigma/Merck, T4174-100 ML, v/v in PBS) was added and incubated for 30–90 s to detach the cells. Cells were collected in their corresponding culture medium and adjusted to a final concentration of 1 × 10^6^ cells/mL. The sensor was placed on a clean layer of Parafilm M (Huberlab, 15.1550.03) in a Petri dish. The tip of the sensor containing the cantilever was layered with a drop of the cell suspension and incubated for 1.5 h at 37 °C and 5% CO_2_. Subsequently, the cell suspension was removed, and the sensor was gently washed with PBS. The cantilever was transferred into the measurement chamber containing prewarmed cell culture medium (Figure 1c). The cell attachment quality was assessed by microscopy.

### Resistell Nanomotion-Based DST Experimental Setup

DST was performed using Resistell nanomotion devices.^10^ The experimental setup was housed within a CO_2_-supplied incubator (Eppendorf, CellXpert C170i) to maintain optimal growth conditions. Nanomotion recordings were acquired at a sampling rate of 60,000 samples per second and consisted of three distinct phases: (i) a short *Blank* phase to establish the baseline deflections of bare functionalized cantilevers in cell culture medium, (ii) a 2 h *Medium* phase, and (iii) an up to 6 h *Drug* phase. The recorded signal represents subnanometer micromechanical transducer deflections generated by living cells.

To minimize external vibrations, the incubator was equipped with a custom-made rigid shelf that supported two measurement heads installed on the active vibration damping units. All control cables were gastight sealed within the incubator’s customized pass-through to ensure operator safety and maintain a constant CO_2_ concentration throughout the experiment.

Micromechanical sensors used in the study were NPO-10.B cantilevers (Bruker), composed of nonconductive silicon nitride. The top surface, where cell attachment occurred, remained uncoated, while the bottom surface was fully coated with reflective gold (Au) to enable the optical detection of nanomotion. Each triangular-shaped cantilever featured two prongs, each with a surface area of 4,800 μm^2^, and a triangular tip with a surface area of 9,100 μm^2^, yielding a total available attachment area of 18,700 ± 500 μm^2^. The narrowest region of the cantilever measured 44 μm in width, which exceeded the average diameters of HeLa and SW480 cells (approximately 17 μm), ensuring a sufficient surface area for cell attachment.

### ML and Development of Classification Models

The device detects nanomotion signals generated by the activity of living cells. In the proposed method, each vibration signal obtained from elastic microbeams is analyzed to extract the temporal evolution of the spectral quantiles. These quantiles are computed analogously to the power spectral density (PSD), employing a modified version of Welch’s method. Rather than averaging periodograms across predefined frequency intervals, the method estimates quantiles of the power distribution, yielding a probabilistic representation of the spectral power as a function of frequency.

By tracking the evolution of these spectral quantiles over time (with a temporal resolution of approximately 5 min), a three-dimensional representation of the signal is constructed. This representation spans the quantile level, frequency, and time, enabling a more nuanced characterization of signal dynamics.

The temporal evolution of the spectral quantiles is then leveraged to extract potential input features for classification. Various statistical descriptors are computed over extended time intervals, including their absolute values, differences, and ratios. These features are designed to be robust to variations under the measurement conditions.

To identify the most informative features, a forward sequential feature selection strategy is employed. Initially, single-variable models are trained for all candidate features, and the model yielding the highest classification accuracy is selected. In subsequent iterations, additional previously unused features are incrementally added to the model. After each addition, performance is re-evaluated and the process continues until no further improvement in model accuracy is observed.

Model performance is evaluated using a predefined validation set. Final estimates of accuracy and sensitivity are obtained using an independent test set.

### Statistical Analysis

Graphs were generated with GraphPad Prism v10.3.1. Statistical analyses were performed using one-way ANOVA with multiple comparisons (Tukey’s multiple comparison test). For the graphs presented in the figures, significance was denoted as nonsignificant (ns) (*p* > 0.05); *p < 0.05; **p < 0.01; ***p < 0.001; ****p < 0.0001. The number of independent biological replicates is indicated as n in the figure legends.

## Supplementary Material



## Notes

Suppl. Figure S1: The doxorubicin structure; Suppl. Figure S2: Nanomotion devices in CO_2_ incubator; Suppl. Figure S3: Quantitative analysis of cell attachment on glass surfaces functionalized with different linking agents reveals good cell attachment on PDL + laminin + fibronectin; Suppl. Figure S4: Functionalization of glass surfaces with PDL + laminin + fibronectin enables stable cell attachment; Suppl. Figure S5: Additional performance metrics for classifying SW480 colon cancer cells as doxorubicin-treated or untreated using nanomotion-derived features; Suppl. Figure S6: Determination of IC_50_ for A2780 and A2780ADR after 24 h doxorubicin treatment; Suppl. Figure S7: Time analysis to determine the optimal duration of the drug phase in silico; Suppl. Figure S8: Additional performance metrics for classifying A2780 and A2780ADR ovarian cancer cells as doxorubicin-susceptible or -resistant using nanomotion-based features (PDF)

## Abbreviations

A2780: human ovarian cancer cell line (chemotherapy-sensitive)
A2780ADR: doxorubicin-resistant variant of A2780 (ADR = adriamycin-resistant)
ANOVA: analysis of variance
DST: drug susceptibility test
DMEM: Dulbecco’s Modified Eagle Medium
DOX: doxorubicine
ECM: extracellular matrix
EDTA: ethylenediaminetetraacetic acid
FCS: fetal calf serum
FDA: U.S. Food and Drug Administration
HeLa: Henrietta Lacks cell line (cervical cancer)
MAPTrix: matrix for advanced physiological tissue-like reconstruction
MFI: mean fluorescence intensity
ML: machine learning
PBS: phosphate-buffered saline
PDL: poly-d-lysine (PDL)
PSD: power spectral density
Abs: absorbance
RPMI: Roswell Park Memorial Institute medium
SLED: superluminescent light-emitting diode
SW480: human colon cancer cell line (derived from colorectal adenocarcinoma)
