# Supplementary material for: Nanomotion-Based Drug Sensitivity Prediction in Ovarian and Colon Cancer Cell Lines Using Machine Learning
Source: ACS Pharmacol Transl Sci. 2025 Aug 18;8(9):3149–59. doi: 10.1021/acsptsci.5c00312 (PMC12441847; doi:10.1021/acsptsci.5c00312)
Supplement: Supplementary file 1 [file pt5c00312_si_001.pdf]

Supporting Information

Nanomotion-Based Drug Sensitivity Prediction in Ovarian and Colon Cancer Cell Lines Using Machine Learning

Katja Fromm[1], Jan Winnicki[1], Grzegorz Jozwiak[1], Gino Cathomen[1], Christine Wagner[1], Marta Pla Verge[1], Eric Delarze[1], Michał Świątkowski[1], Grzegorz Wielgoszewski[1], Maria Ines Villalba[2,]3, Laura Munch[1], Sandor Kasas[2,3] , Danuta Cichocka[1] and Alexander Sturm[1]*

[1] Resistell AG, Hofackerstrasse 40, 4132 Muttenz, Switzerland
[2] Laboratory of Biological Electron Microscopy (LBEM), École Polytechnique Fédérale de Lausanne (EPFL), Université de Lausanne, 1015 Lausanne, Switzerland
[3] Centre Universitaire Romand de Médecine Légale (UFAM), Université de Lausanne, 1000 Lausanne, Switzerland

*corresponding author: Alexander Sturm, https://orcid.org/0000-0002-3818-0428, alex.sturm@resistell.com

Table of Contents for Supplementary Information

Suppl. Figure S1: The Doxorubicin structure S-3
Suppl. Figure S2: Nanomotion devices in CO_2 incubator S-4
Suppl. Figure S3: Quantitative analysis of cell attachment on glass surfaces functionalized with different linking agents reveals good cell attachment on PDL + laminin + fibronectin S-5
Suppl. Figure S4: Functionalization of glass surfaces with PDL + laminin + fibronectin enables stable cell attachment S-6
Suppl. Figure S5: Additional performance metrics for classifying SW480 colon cancer cells as doxorubicin-treated or untreated using nanomotion-derived features S-7
Suppl. Figure S6: Determination of IC_{50} for A2780 and A2780ADR after 24h doxorubicin treatment S-8
Suppl. Figure S7: Time Analysis to Determine the Optimal Duration of the Drug Phase in silico S-9
Suppl. Figure S8 Additional performance metrics for classifying A2780 and A2780ADR ovarian cancer cells as doxorubicin-susceptible or -resistant using nanomotion-based features S-10

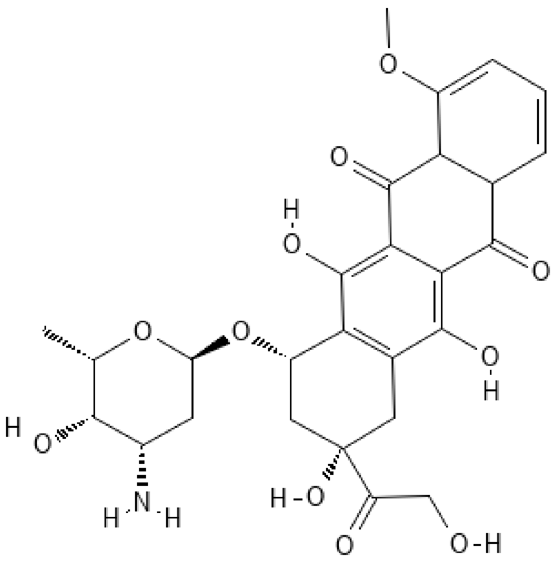

Suppl. Figure S1: The Doxorubicin structure (DOX, $C_{27}H_{29}NO_{11}$) consists of a tetracyclic anthracycline aglycone (doxorubicinone) linked to daunosamine. The structure was drawn using the PubChem Sketcher V2.4.

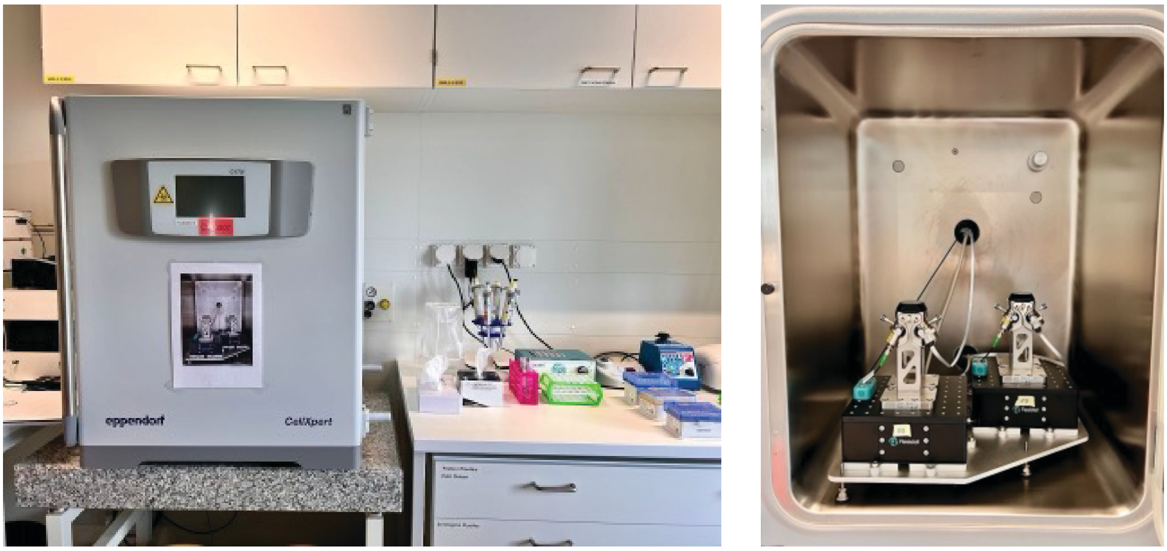

Suppl. Figure S2: Nanomotion devices in CO_2 incubator maintaining stable temperature of 37ºC and 5% CO_2 concentration.

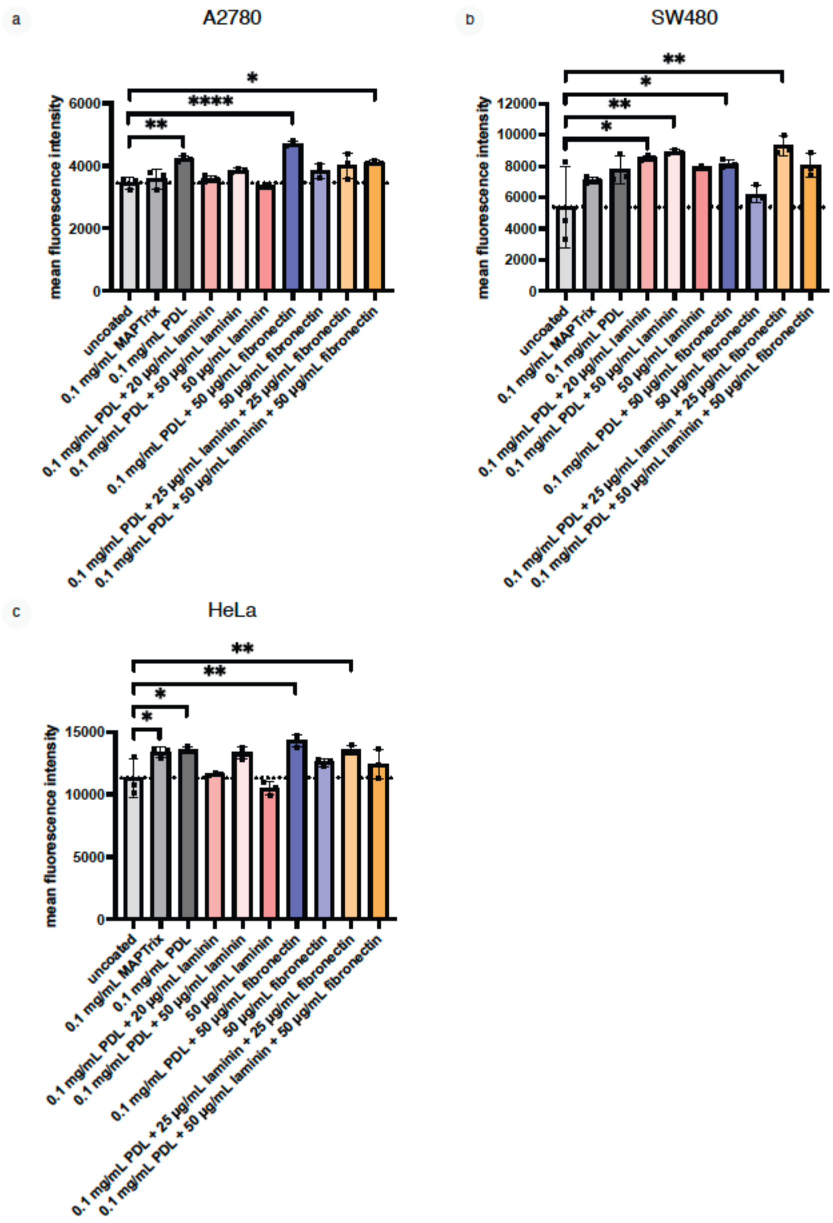

Suppl. Figure S3: Quantitative analysis of cell attachment on glass surfaces functionalized with different linking agents reveals good cell attachment on PDL + laminin + fibronectin

The ovarian cancer cell line A2780 was cultured in RPMI + 10% FCS, while the colon cancer cell line SW480 and HeLa cells were cultured in DMEM + 10% FCS. All cells were maintained at 37°C and 5% CO_2. A total of 5×10^4 cells were seeded per well in a 96-well plate, incubated for 1 hour, and subsequently washed with PBS to remove non-attached cells. After washing, 100 µL of fresh cell culture medium was added to each well, supplemented with 10 µL of 0.05% resazurin (w/v in H_2O). To assess cellular activity, the mean fluorescence intensity (MFI) of the resazurin reduction product, resorufin, was measured every 20 minutes for four hours using the FLUOstar Omega plate reader.

Panels (a–c) show MFI readings over time for: (a) A2780 cells, (b) SW480 cells, and (c) HeLa cells. All data represent mean ± SD of technical triplicates of three independent experiments. Statistical analysis was performed using one-way ANOVA with Tukey's multiple comparison test. Statistical significance is indicated as follows: *$p < 0.05$, **$p < 0.01$, ***$p < 0.001$, ****$p < 0.0001$.

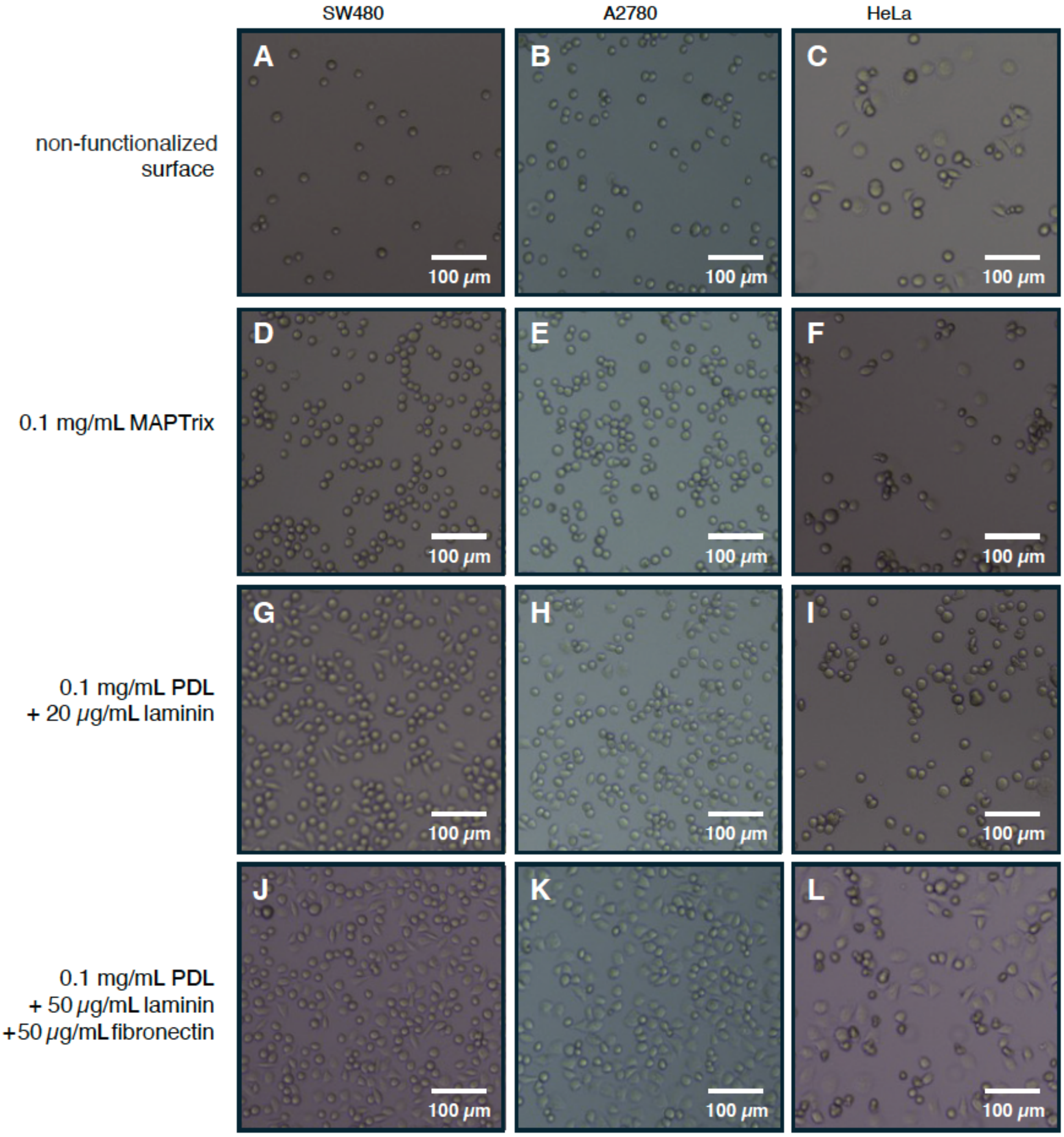

Suppl. Figure S4: Functionalization of glass surfaces with PDL + laminin + fibronectin enables stable cell attachment

The ovarian cancer cell line A2780 was cultured in RPMI + 10% FCS, while the colon cancer cell line SW480 and HeLa cells were cultured in DMEM + 10% FCS. All cells were maintained at 37°C and 5% CO_2. A total of 5×10^4 cells were seeded per well in a 96-well plate, incubated for 1 hour, and washed with PBS to remove non-attached cells. After washing, 100 µL of fresh cell culture medium was added to each well, and cell attachment was assessed by microscopy. Panels (A–L) display representative images of cell attachment under different surface conditions: (A–C) Non-functionalized glass surface: (A) SW480 cells, (B) A2780 cells, and (C) HeLa cells. (D–F) Surface functionalized with 0.1 mg/mL MAPTrix: (D) SW480 cells, (E) A2780 cells, and (F) HeLa cells. (G–I) Surface functionalized with 0.1 mg/mL poly-D-lysine (PDL) + 20 µg/mL laminin: (G) SW480 cells, (H) A2780 cells, and (I) HeLa cells. (J–L) Surface functionalized with 0.1 mg/mL PDL + 50 µg/mL laminin + 50 µg/mL fibronectin: (J) SW480 cells, (K) A2780 cells, and (L) HeLa cells. All data represent representative results from of independent experiments.

a

1 Feature

	Training	Validation	Testing
F1	85.7%	75.0%	100.0%
Precision	90.0%	100.0%	100.0%
Recall	81.8%	60.0%	100.0%

b

2 Features

	Training	Validation	Testing
F1	95.2%	88.9%	92.3%
Precision	100.0%	100.0%	100.0%
Recall	90.9%	80.0%	85.7%

c

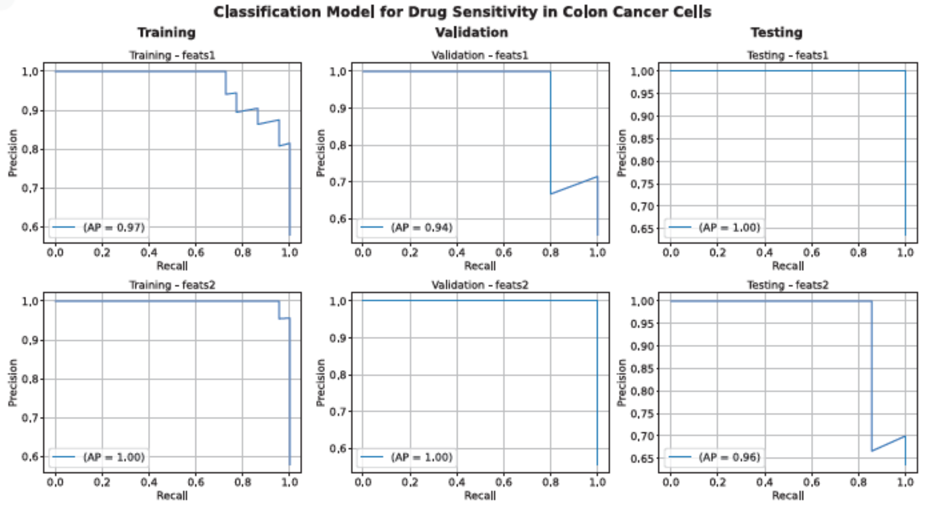

Suppl. Figure S5: Additional performance metrics for classifying SW480 colon cancer cells as doxorubicin-treated or untreated using nanomotion-derived features. The dataset comprised 34 treated (positive) and 24 untreated (negative) cells, split into training (22 treated / 16 untreated), validation (5 treated / 4 untreated), and test sets (7 treated / 4 untreated). Cells were recorded for two hours in standard medium followed by two hours with 32 µM doxorubicin. Models were trained using either one (a) or two features (b) extracted from the nanomotion data, and evaluated using precision, recall (treated), and F1 score across all dataset partitions. The results show consistently high predictive performance. (c) Precision–recall curves indicate that all models achieved average precision (AP) scores well above the random classifier baseline of 0.64 in the test set, with values approaching 1.0, suggesting strong discriminatory power. Precision represents the ratio of true positive predictions to all positive predictions. Recall indicates the ratio of true positive predictions to all actual positive cases (in this case, "treated"). The F1 score represents their harmonic mean.

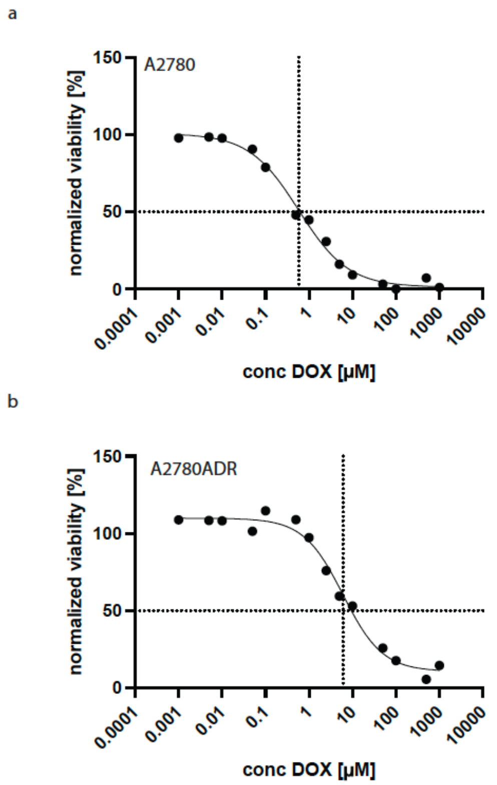

Suppl. Figure S6: Determination of IC_{50} for A2780 and A2780ADR after 24h doxorubicin treatment
The ovarian cancer cell lines A2780 (a) and A2780ADR were cultured in RPMI + 10% FCS at 37°C and 5% CO_2. A total of 2×10^4 cells were seeded per well and incubated for 24 hours. Increasing concentrations of doxorubicin were then added, followed by an additional 24-hour incubation. Subsequently, the medium was supplemented with 0.005% resazurin (w/v in H_2O) to assess cell viability. The absorbance of resazurin (600 nm) and its reduced product, resorufin (570 nm), was measured every 20 minutes for four hours using the FLUOstar Omega plate reader. Panels (a-b) depict the relative cell viability of (a) A2780 cells and (b) A2780ADR cells. Each dot represents the mean of three technical replicates, with a linear regression fitted to the data. A dotted line on the x-axis indicates the IC_{50} value for each cell line. All data are representative of three independent experiments.

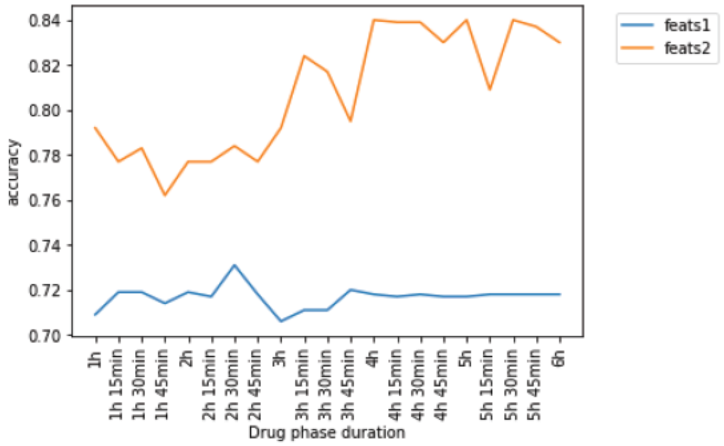

Suppl. Figure S7: Time Analysis to Determine the Optimal Duration of the Drug Phase in silico
To determine the most suitable drug phase duration for model development, we analyzed nanomotion recordings from A2780 and A2780ADR cells exposed to doxorubicin (0.01, 0.1, or 10 µM). Each experiment consisted of a 2-hour medium phase followed by a 6-hour drug phase, with features extracted exclusively from the drug phase. Performance was evaluated across different drug phase durations, considering models trained on one or two features. The selected duration was 4 hours and 15 minutes, with data recorded at 15-minute intervals. All subsequent models were developed using this duration and time intervals. At 4 hours and 15 minutes, performance plateaued at approximately 84% accuracy, suggesting that the final 1 hour and 45 minutes of the recording did not contribute to model improvement and was excluded from further development. The figure presents the performance results based on cross-validation.

a

1 Feature

	Training	Validation	Testing
F1	79.2%	76.9%	66.7%
Precision	75.0%	71.4%	80.0%
Recall	84.0%	83.3%	57.1%

b

2 Features

	Training	Validation	Testing
F1	90.6%	83.3%	83.3%
Precision	85.7%	83.3%	100.0%
Recall	96.0%	83.3%	71.4%

c

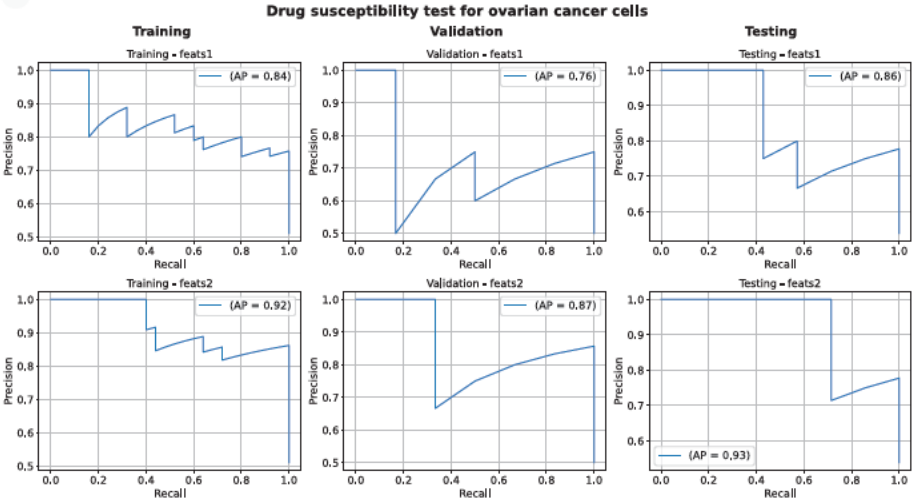

Suppl. Figure S8 Additional performance metrics for classifying A2780 and A2780ADR ovarian cancer cells as doxorubicin-susceptible or -resistant using nanomotion-based features. The dataset consisted of 38 susceptible (positive) and 36 resistant (negative) cells, divided into training (25 susceptible / 24 resistant), validation (6 susceptible / 6 resistant), and test sets (7 susceptible / 6 resistant). Nanomotion recordings were acquired over a 6-hour and 15-minute protocol: 2 hours in drug-free medium followed by 4 hours and 15 minutes of treatment with 0.01, 0.1, or 10 µM doxorubicin. Models trained on one (a) or two (b) extracted features were evaluated using precision, recall, and F1 score. (c) The precision–recall curves show that the average precision (AP) consistently exceeds the random baseline of 0.54 and approaches 0.9, indicating strong predictive accuracy. Precision represents the ratio of true positive predictions to all positive predictions (in this case, “doxorubicin susceptible”). Recall indicates the ratio of true positive predictions to all actual positive cases. The F1 score represents their harmonic mean.
